# Synthesis and Antioxidant Activity of Silver Nanoparticles Using the *Odontonema strictum* Leaf Extract

**DOI:** 10.3390/molecules27103210

**Published:** 2022-05-17

**Authors:** Lokadi Pierre Luhata, Christian Nanga Chick, Natsuki Mori, Kunihito Tanaka, Hiroshi Uchida, Takashi Hayashita, Toyonobu Usuki

**Affiliations:** Department of Materials and Life Sciences, Faculty of Science and Technology, Sophia University, 7-1 Kioicho, Chiyoda-ku, Tokyo 102-8554, Japan; luhatagot@yahoo.fr (L.P.L.); chicknanga0@gmail.com (C.N.C.); monakaarisu@eagle.sophia.ac.jp (N.M.); tanaka@sophia.ac.jp (K.T.); uchidah@sophia.ac.jp (H.U.); ta-hayas@sophia.ac.jp (T.H.)

**Keywords:** *Odontonema strictum*, silver nanoparticles, phytoconstituents, antioxidants

## Abstract

The aqueous extract of the leaves of *Odontonema strictum* (OSM) is used in folk medicine for its antihypertensive properties, and it contains a wide range of secondary metabolites, mostly polyphenols such as verbascoside and isoverbascoside, which could play a major role in the preparation of silver nanoparticles. In this study, we aimed to prepare AgNPs for the first time using the OSM leaf extract (OSM-AgNPs) to investigate their free radical-scavenging potency against 1,1-diphenyl-2-picrylhydrazyl (DPPH) and hydrogen peroxide (H_2_O_2_). Dynamic light scattering (DLS), UV/Vis, Fourier-transform infrared spectroscopy (FTIR), scanning electron microscopy (SEM), energy-dispersive X-ray (EDX), and X-ray photoelectron spectroscopy (XPS) were used to characterize the OSM-AgNPs. With a size around 100 nm and a ζ-potential of −41.1 mV, OSM-AgNPs showed a good stability and a better colloidal property due to electrostatic repulsion and the dispersity. The strong absorption peak at 3 keV in the EDX spectra indicated that silver was the major constituent. Additionally, the existence of silver atoms was confirmed by the Ag 3d_5/2_ peak around 367 eV in the XPS spectra. IC_50_ values of 116 μg/mL and 4.4 μg/mL were obtained for the scavenging activities of DPPH and H_2_O_2_, respectively. The synthetic OSM-AgNPs can be further exploited as potential antioxidant agents.

## 1. Introduction

In the last 20 years, nanotechnology has received great attention due to its large and attractive areas of research [1,2]. In fact, nanoparticles (NPs) of various metals, including noble ones, such as silver, gold, platinum, copper, zinc, titanium, and magnesium, have gained considerable attention for diverse applications due to their multifunctional theranostic abilities [3]. Their small size (<100 nm) and large surface area-to-volume ratio, as well as their magnetic, chemical, and mechanical properties, have made them candidates for novel applications in the biomedical field as antibiotic, antioxidant, and anticancer agents [4,5]. Many other innovative applications such as catalysis, fuel cells, magnetic data storage, and solar cells are reported [6]. NPs can be synthesized using various approaches including chemical, physical, and biological approaches [7]. However, most of these methods are relatively expensive and involve the use of toxic, hazardous chemicals, which may pose potential environmental and biological risks. In addition, these methods require high energy and space [8,9].

Among metal NPs, AgNPs are gaining enormous interest in the research community due to their wide scope of application in microbiology, chemistry, food technology, cell biology, pharmacology, and parasitology. AgNPs are reported to have potential anticancer, antimicrobial, antioxidant, antifungal, anti-inflammatory, antiviral, antiangiogenetic, and antiplatelet activities [10,11,12,13]. The morphology of AgNPs is the deciding factor in their physical and chemical properties. The slow and regulated release of silver from silver nanoparticles is one of the most striking advantages of these nanoparticles when compared with bulk metals and their salts [5,14,15,16].

Recently, plant-mediated synthesis of AgNPs has acquired great attention due to its lower toxicity, cost efficiency, eco-friendliness, and short operation time [17]. Generally, AgNPs are produced by the reduction of silver ions to metal nanoparticles using different biomolecules such as flavonoids, ketones, aldehydes, tannins, carboxylic acids, phenolics, and proteins of plant extracts which act as reducing, stabilizing, and capping agents in the conversion of metal ions to metal nanoparticles [18].

The aqueous extract of *Odontonema strictum* (OSM) is used in Burkina Faso (western Africa) by traditional medicine practitioners against hypertension [19]. In our previous work, the phytochemical screening (Table 1) of the MeOH–DCM leaf extract led to the isolation of stigmasterol and beta-sitosterol [20,21]. Using reversed-phase high-performance liquid chromatography (RP-HPLC), the chromatogram of the leaf extract confirmed that OSM biosynthesizes a large spectrum of secondary metabolites (Figure S1). The antibacterial potency of β-sitosterol against *Staphylococcus aureus* was evaluated, and this phytosterol exhibited a low effect [22].

More recently, we isolated, characterized, and evaluated the antioxidant properties of verbascoside and isoverbascoside from the leaf extract of OSM [23]. These two phenylpropanoid glycosides, highly soluble in water, exhibited good antioxidant properties in laboratory conditions and could be key to understanding the antihypertensive activity of OSM. The phytochemical and bioactivity information above led to the current study aimed at the investigation of the plant-mediated synthesis of silver nanoparticles (OSM-AgNPs) using OSM leaf extract and an in vitro evaluation of their antioxidant potential. In fact, molecules and nanoparticles with antioxidant properties might play a key role in preventing free radical-induced diseases such as cancer, rheumatoid arthritis, inflammation, and hypertension. This is the first time that silver nanoparticles have been synthesized and characterized from this plant.

## 2. Results and Discussion

**Figure 1:**
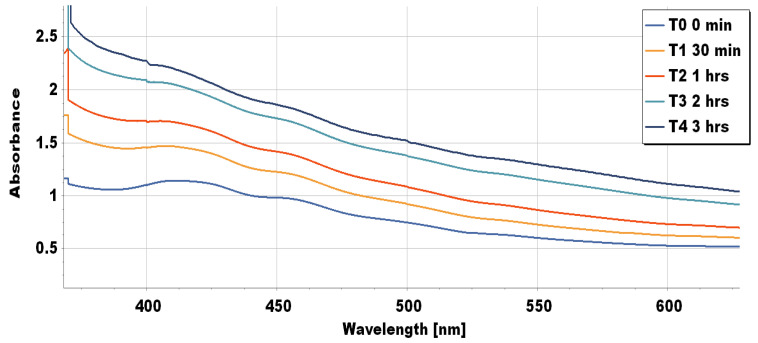
The presence of an absorbance peak around 420 nm clearly indicates the formation of OSM-AgNPs in the solution, while the intensity of the SPR band increased with reaction time, indicating the formation of the nanoparticles.

OSM-AgNPS were synthesized from the aqueous leaf extract of OSM after adding silver nitrate solution. The phytochemicals previously mentioned acted as catalysts for the reduction of silver ions to silver. In fact, the change in color from yellow to brown after 3 h of constant stirring confirmed the presence of metal nanoparticles (Figure S2). The monitoring of the process with a UV/Vis instrument revealed the presence of a sharp peak around 420 nm similar to previous studies, due to the surface plasmon resonance (SPR) of electrons present on the surface of the synthesized nanoparticles (Figure 1) [7,24]. In addition, the intensity of the SPR band increased with reaction time, indicating the synthesis of the OSM-AgNPs [4].

**Figure 2:**
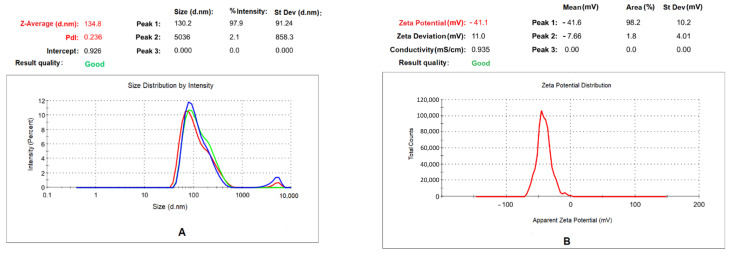
Nanoparticle size measurements: (**A**) size and PDI (red, green, and blue traces corresponding to individual measurements); (**B**) ζ-potential of the OSM-AgNPs.

The dynamic light scattering (DLS) technique was used to measure the hydrodynamic particle size distribution, polydispersity index (PDI), and surface charge (ζ-potential) of the OSM-AgNPs. The average size of the synthesized OSM-AgNPs was found to be approximately 135 nm with a low polydispersity index of 0.2 (Figure 2A) and a ζ-potential of −41.1 mV (Figure 2B). It is good to point out the fact that the hydrodynamic size includes the hydration layer on the surface of AgNPs. Thus, the observed 130 nm is larger than the size measured by scanning electron microscopy (SEM) images [4]. In addition, a nanoparticle size value below 150 nm and PDI values around 0.3 are adequate for uptake by cells [25]. Concerning the stability of the OSM-AgNPs, the ζ-potential of −41.1 mV reveals that they have a good stability and a better colloidal property due to electrostatic repulsion and dispersity. The negative charge indicates that the negatively charged functional groups from the plant extract probably contribute to the colloidal stability of these nanoparticles [4,26].

**Figure 3:**
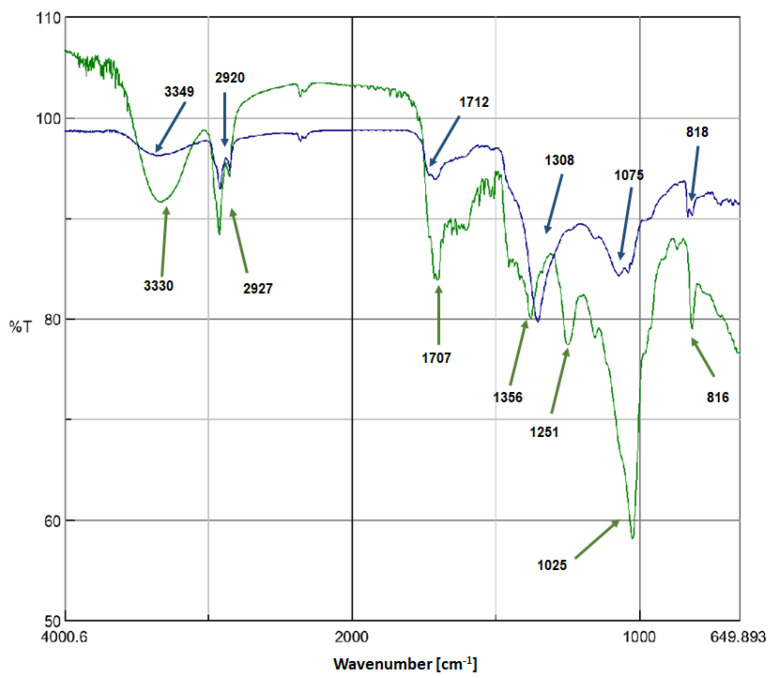
FTIR spectra of the OSM leaf extract (green) and OSM-AgNPs (blue).

Fourier-transform infrared (FTIR) spectroscopy was used to identify the major vibrations involved in the reduction, stabilizing, and capping of OSM-AgNPs, as shown in Figure 3. The FTIR spectrum of the OSM leaf extract showed absorption peaks at 3330, 2927, 2851, 1707, 1308, 1251, 1025, and 816 cm^−1^. Meanwhile, the FTIR spectrum of OSM-AgNPs showed major absorption peaks at 3349, 2920, 2853, 1712, 1356, 1075, and 818 cm^−1^.

The analysis of the spectra above shows some minor and major shifts of the peaks which can be reasonably ascribed to the reduction, capping, and stabilization of the OSM-AgNPs. A shift was observed for the peak at 3330 cm^−1^ to a higher wavelength of 3349 cm^−1^, probably due to the involvement of the O–H or N–H stretching of phenolic compounds present in the leaf extract, which may interact with the silver ions. The absorption peaks at 2927 and 2920 cm^−1^ were due to the C–H stretching of methylene groups or aliphatic groups, representing the characteristic peak of triterpenoid saponins. The shift from 1707 cm^−1^ to 1712 cm^−1^ could confirm the involvement of C=O vibrations. Moreover, the shift at 1379 cm^−1^ to 1356 cm^−1^ was possibly due to the –C–O stretching of phenol or tertiary alcohols. The disappearance of the peak at 1251 cm^−1^ may have been due to the partial break of C–O bonds at 80 °C during the synthesis process. Bands at 816 and 818 cm^−1^ were probably due to O–H stretching or C–S stretching or the involvement of aliphatic chloro compounds. These assignments are in agreement with many reports regarding the synthesis of AgNPs [27,28,29,30,31,32].

**Figure 4:**
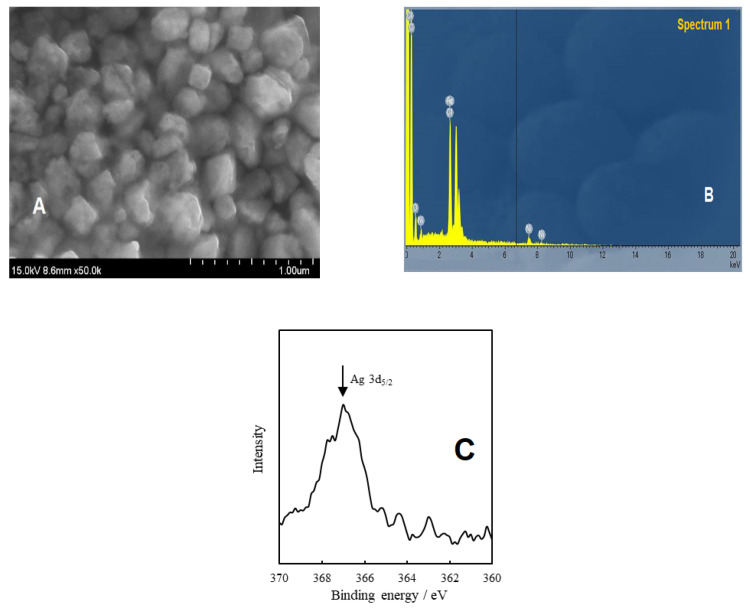
SEM image and EDX and XPS spectra of OS-AgNPs. (**A**) The morphology of the OSM-AgNPs was found to be irregularly granulated and highly aggregated, with some presenting a cubic shape. (**B**) The strong absorption peak at 3 keV confirmed the presence of silver. (**C**) The observed peak of Ag 3d_5/2_ around 367 eV confirmed the existence of silver atoms.

The SEM technique was employed to visualize the size and the morphology of the synthesized OSM-AgNPs. The prepared nanoparticles were found to be in polymorphic shapes and highly aggregated, probably caused by dehydration during the preparation of samples for SEM analysis (Figure 4A) [33]. If the observed hydrodynamic size (130 nm) is generally larger than the size measured from SEM, we can conclude that the synthesis of AgNPs was successful [4]. In fact, the analysis of the SEM image revealed that OSM-AgNPs had a size around 100 nm, in agreement with the DLS results.

Additionally, the strong absorption peak at 3 keV in the EDX spectra (Figure 4B) indubitably confirmed that silver was the major constituent [4]. According to Francesco Porcaro and coworkers, the Ag3d_5/2_ component at about 367 eV corresponds to unperturbed metallic silver, as expected for atoms in the NP core [34]. In fact, the Ag 3d_5/2_ electrons in zero-valency Ag, monovalent Ag_2_O, and divalent AgO were reported to have binding energies in the ranges of 367.9–368.3, 367.7–368.4, and 367.3–368.1 eV, respectively, by XPS analysis (Figure 4C) [35].

Recent reports have confirmed the substantial antioxidant potential of silver nanoparticles prepared with plant extracts such as aqueous leaf and fruit extracts [36,37]. In fact, the capping of silver with phytochemicals of plant extract is assumed to be responsible for antioxidant activity exhibited by AgNPs. Many studies showed that AgNPs capped by aqueous leaf extract exhibited higher antioxidant potential than the leaf extracts [38,39].

Table 2 shows that OSM-AgNPs were less able to scavenge DPPH radicals (IC_50_ = 116 μg/mL) and H_2_O_2_ (IC_50_ = 4.41 μg/mL) compared to the leaf extract (IC_50_ = 91 μg/mL and = 3.73 mg/mL) and the control (IC_50_ = 0.31 mg/mL and 2.80 mg/mL) (Figure S3). The results reveal clearly that the protocol used for the preparation of OSM-AgNPs could have been the cause of the reduction in activity. In these two assays, the leaf extract appeared to be more potent than the synthesized nanoparticles, implying that the use of 80 °C in the process for 3 h may have led to the destruction of some bioactive phytochemicals. Although stable synthesis of plant-extract mediated AgNPs can occur in a diverse temperature range (25 to >40 °C) [40], toom temperature is preferable as this temperature range triggers the formation of spherical AgNPs with single surface plasmon at low wavelengths.

## 3. Materials and Methods

### 3.1. Solvents and Reagents

H_2_O in HPLC grade was purchased from Kishida Chemical (Osaka, Japan). 1,1-Diphenyl-2-picrylhydrazyl (DPPH) was purchased from TCI (Tokyo, Japan). Hydrogen peroxide (H_2_O_2_) was purchased from Kanto Chemical (Tokyo, Japan). Silver nitrate was purchased from Fujifilm Wako Pure Chemical Corporation (Osaka, Japan).

### 3.2. Instruments

UV analysis: A JASCO (Tokyo, Japan) V-730BIO spectroscope was used. Spectra were recorded in the range of 190–700 nm with 1 nm resolution.

Infrared analysis: A JASCO FT/IR-4100 (Fourier-transform infrared) spectroscope was used to analyze samples with ATR. 

Dynamic light scattering (DLS): Size and zeta potential measurements were carried out at 25 °C using a Zetasizer Nano ZS (Malvern Instruments Ltd., Malvern, Worcestershire, UK).

Film coating: A JEOL JEC-3000 was used.

Scanning electron microscopy (SEM): A Hitachi (Tokyo, Japan) SU8000 was used.

Energy-dispersive X-ray (EDX): A Hitachi SU8000 with Horiba (Kyoto, Japan) EMAX x-act analyzer was used.

X-ray photoelectron spectroscopy (XPS): The existence of Ag atoms was investigated by XPS (Versa Probe II, ULVAC-PHI, Inc.; Kanagawa, Japan). The X-ray source was monochromatized Al Kα radiation. The takeoff angle was 45°. The binding energies of XPS spectra were corrected with the C1s peak position at 284.6 eV.

### 3.3. Plant Materials

#### 3.3.1. Collection and Taxonomy

Plant specimens (leaves, stems, and roots) were collected in Kinshasa (DRC) (June–July 2021) and dried in the shade at room temperature for 2 weeks. The collected plant materials were compared to a previous voucher specimen PLL3 identified by Dr. Chuba, deposited in the Herbarium of the Department of Biological Sciences at the University of Zambia, and later sent to the Usuki Laboratory at Sophia University.

#### 3.3.2. Preparation and Extraction for the Crude Extract

Dried samples (365 g of leaves) were extracted three times with a mixture of 1 L of methanol (MeOH) and 1 L of dichloromethane (DCM) at room temperature for 72 h (3×, 2 L). The MeOH–DCM solution was filtered through a cotton hood and evaporated under a vacuum at <40 °C to afford 47.45 g of crude extract (13% of yield).

#### 3.3.3. Synthesis of OSM-AgNPs

The synthesis of silver nanoparticles was carried out using the leaf extract of *O. strictum* prepared in the previous step. To 10 mL of leaf extract (4 mg/mL), 90 mL of 1 mM aqueous silver nitrate solution was added, and the mixture was heated at 80 °C for 3 h with stirring. The formation of the AgNPs was preliminarily detected by the change in color from yellow to dark brown. The green-synthesized nanoparticles were separated using centrifugation at 15,000× *g* for 20 min. This process was repeated thrice to get rid of free silver associated with Cp-AgNPs. The final, green-synthesized silver nanoparticles were freeze-dried and then stored at 4 °C until further use.

### 3.4. Bioassays

#### 3.4.1. 1,1-Diphenyl-2-Picrylhydrazyl (DPPH) Assay

The method described by Alhage et al. (2018) [41] was used with few modifications. Concentrations of samples (0.0001, 0.001, 0.01, 0.1, and 0.5 mg/mL) were prepared in methanol. To 1 mL of the sample was added 1 mL of DPPH (0.16 mM in MeOH), which was kept in the dark for 30 min; then, the absorbance was read at 517 nm. The efficacy of scavenging DPPH radicals was calculated using Equation (1).

% Inhibition = ((A control − A sample))/(A control) × 100,(1)

where A control is the absorbance of the control (DPPH solution without sample), and A sample is the absorbance of the test sample (DPPH solution with test sample) at 517 nm. Triplicate measurements were carried out. Vitamin C was used as a positive control.

#### 3.4.2. Hydrogen Peroxide (H_2_O_2_) Assay

The protocol described by Aryal et al. (2019) [42] was used with slight modifications. Concentrations of samples (0.0001, 0.001, 0.01, 0.1, and 0.5 mg/mL) were prepared in distilled water containing 20% of MeOH. To 2 mL of H_2_O_2_ 20 mM prepared from PBS (pH = 7.4) was added 1 mL of the sample and incubated for 10 min. The absorbance was read at 230 nm. Ascorbic acid was used as a standard. The efficacy of scavenging H_2_O_2_ radicals was calculated using Equation (2).

% scavenging H_2_ O_2_ = [(A_0_ − A_1_)/A_1_] × 100, (2)

where A_0_ is the absorbance of the control (PBS solution without H_2_O_2_), and A_1_ is the absorbance of the extracts or standard.

#### 3.4.3. Statistical Analysis

Analyses were conducted using Excel (2021). Significant differences for comparisons were determined using one-way analysis of variance (ANOVA). The results with a 5% level of confidence were regarded as statistically significant.

## 4. Conclusions

The plant-mediated synthesis of AgNPs appears to be reliable and eco-friendly, requiring less energy, space, and cost [17]. The presence of many various phytochemicals in the leaves of OSM makes it a suitable candidate for the preparation of nanoparticles. In fact, the aqueous fraction contains molecules such as verbascoside, isoverbascoside, iridoids, glucosides, and other polyphenols which may play a major role in the capping or reduction of silver ions. This study showed the ability of the aqueous leaf extract of OSM to effectively prepare AgNPs, which could be used for their antioxidant properties in the management of oxidative stress found in several acute and chronic pathological processes, such as hypertension, cancer, diabetes, and neurodegenerative diseases [43]. In fact, the active secondary metabolites involved in the plant-mediated synthesis of nanoparticles are biocompatible for a wide range of biomedical and nanotechnology-based industries.

## Figures and Tables

**Table 1:** Qualitative phytochemical screening of MeOH-DCM leaf extracts of *O. strictum*.

Phytochemicals	MeOH–DCM Leaf Extract
Tannins	+++
Saponins	+++
Flavonoids	+++
Triterpenoids	+
Glycosides	+
Alkaloids	+/−

+++: strong presence; +: presence; +/−: in very small quantity.

**Table 2:** Free radical-scavenging activities of leaf extract, OSM-AgNPs and ascorbic acid (μg/mL).

Samples	DPPH	H_2_O_2_
Leaf extract	90 ± 18	3.8 ± 0.84
OSM-AgNPs	116 ± 7.5	4.4 ± 0.01
Ascorbic acid	0.31 ± 0.6	2.8 ± 0.03

## Data Availability

The data presented in this study are available on request from the corresponding author.

## Supplementary Materials

The following supporting information can be downloaded at https://www.mdpi.com/article/10.3390/molecules27103210/s1. Figure S1. MeOH-DCM leaf extract chromatogram profile; Figure S2. The change of the color from yellow to dark brown after 3 hours of constant stirring confirmed the synthesis of silver nanoparticles; Figure S3. Scavenging potency of different concentrations of samples on 1,1-diphenyl-2-picrylhydrazyl (A) and hydrogen peroxide (B).
