# Supplementary material for: Synthesis and Antioxidant Activity of Silver Nanoparticles Using the Odontonema strictum Leaf Extract
Source: Molecules. 2022 May 17;27(10):3210. doi: 10.3390/molecules27103210 (PMC9143667; doi:10.3390/molecules27103210)
Supplement: Supplementary file 1 [file molecules-27-03210-s001.zip › molecules-1723844-supplementary.pdf]

Synthesis and Antioxidant Activity of Silver Nanoparticles using the *Odontonema strictum* Leaf Extract

Lokadi Pierre Luhata, Christian Nanga Chick, Natsuki Mori, Kunihito Tanaka, Hiroshi Uchida, Takashi Hayashita, and Toyonobu Usuki*

Department of Materials and Life Sciences, Faculty of Science and Technology, Sophia University, 7-1 Kioicho, Chiyoda-ku, Tokyo 102-8554, Japan

* Corresponding author: t-usuki@sophia.ac.jp (T.U.)

Contents

S2: **Figure S1** (RP-HPLC Chromatogram of the leaf extract)

S3: **Figure S2** (Synthesis of OSM-AgNPs)

S4: **Figures S3** (Activities of DPPH and H_2O_2)

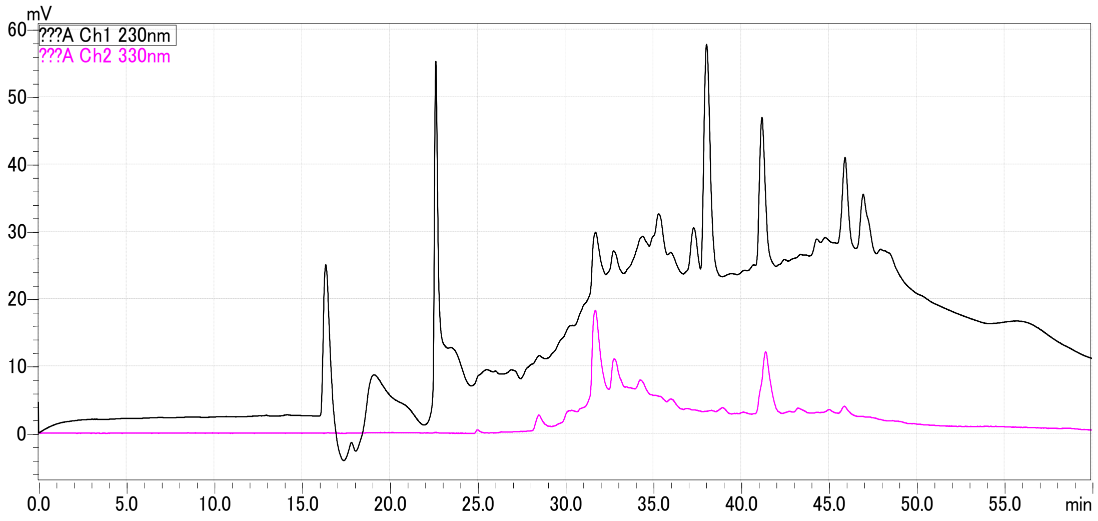

Figure S1. MeOH-DCM leaf extract chromatogram profile. The gradient program: 100% of B to 50 % of B in 5 min and kept in 50 % of B for 5 min, 50% to 0 % of B in 20 min, and 0% to 95% of B in 30 min. Peaks of interest were monitored at l1 230 nm and l2 330 nm. Sample injection: 30 mL; flow rate: 1.0 mL/min.

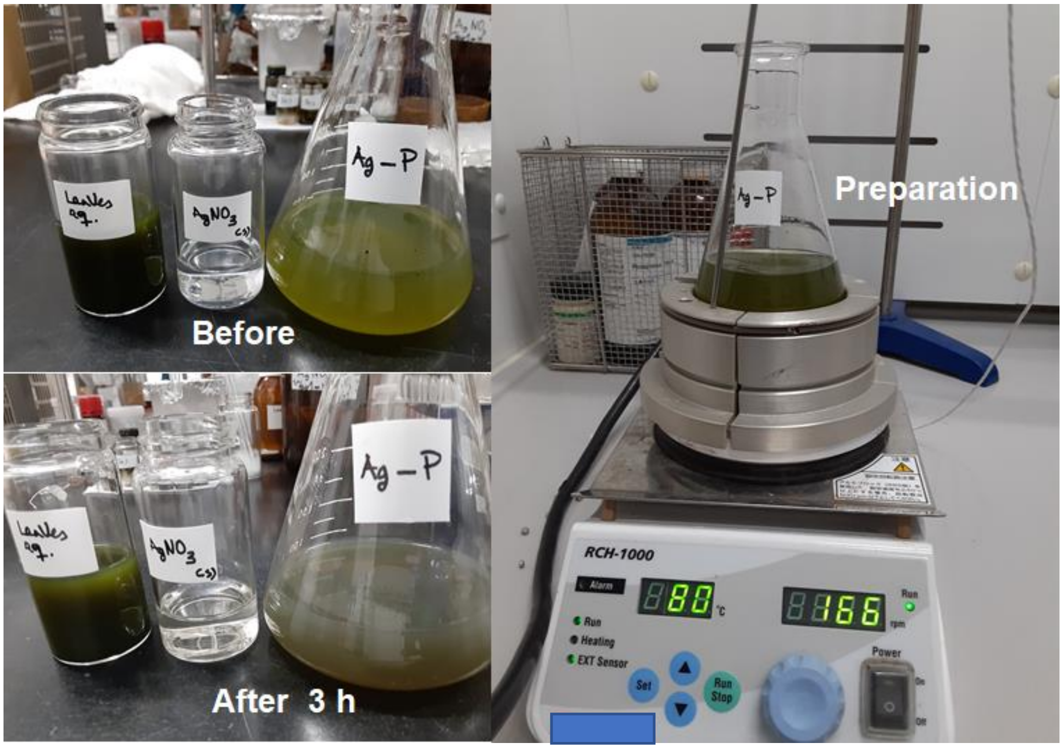

Figure S2. The change of the color from yellow to dark brown after 3 hours of constant stirring confirmed the synthesis of silver nanoparticles.

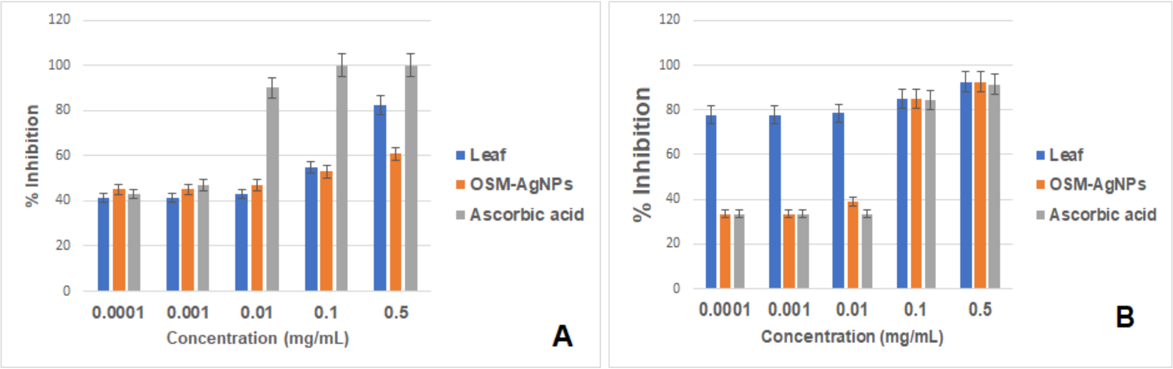

Figure S3. Scavenging potency of different concentrations of samples on 1,1-diphenyl-2-picrylhydrazyl (A) and hydrogen peroxide (B). One-way analysis of variance (ANOVA), multiple comparisons; *P-value signifies < 0.05.
